# Outcomes and linkage to chronic care of HIV exposed infants among health centers and hospitals in Amhara Region, Ethiopia: implications to prevention of mother-to-child transmission of HIV program: a cross sectional study

**DOI:** 10.11604/pamj.2016.24.61.7305

**Published:** 2016-05-13

**Authors:** Zemene Tigabu Kebede, Belaynew Wasie Taye

**Affiliations:** 1 Department of Pediatrics and Child Health, University of Gondar, Ethiopia; 2 Departments of Epidemiology and Biostatistics, Bahir Dar University, Ethiopia

**Keywords:** HIV infection, HIV exposed infant, linkage to chronic care, Northwest Ethiopia

## Abstract

**Introduction:**

Numerous challenges exist in provision of prevention of mother-to-child transmission of HIV (PMTCT) such as linking HIV exposed infants (HEI) and their mothers to chronic cares services, and tackling loss to follow up. Limited evidence exists in Ethiopian setting that explains the persisting high HIV infection rate among HEIs and extent of linkage to chronic care. The study assessed the proportion of HIV infection; children linked to chronic care and determinants of HIV infection among HEI in Northern Ethiopia.

**Methods:**

This institution-based cross-sectional study was conducted in health centers and hospitals of Amhara Region. A total of 484 HEI-mother pairs selected by multistage random sampling were included in the study. Data were collected from PMTCT and anti-retroviral therapy (ART) clinics using pre-tested and structured questionnaires. Quantitative data were entered in Epi Info version 7.0 and exported to SPSS 20.0 for analysis.

**Results:**

A total of 484 mother-infant pairs with a response rate of 92.4% were included in the analysis. About 94.2% of infants and women were linked to chronic care follow-up sometime after the diagnosis. The proportion of HIV infection was 12.4%. Antenatal care attendance had a significant association with HIV infection among HEI (p < 0.0001). Delivering in health institution (p < 0.005), mode of delivery (p < 0.032), and provision of both infant (p < 0.0001) and maternal (p < 0.0001) prophylaxis showed a highly significant association with HIV infection among HIV exposed infants.

**Conclusion:**

Health facilities shall encourage antenatal care that increased institutional delivery, leads to timely initiation and high uptake of PMTCT to reduce the vertical transmission of HIV infection and meet national targets.

## Introduction

In Ethiopia, HIV prevalence among women aged 15-49 years is 1.9 percent and increases with age to a peak of 3.7 percent at age 30-34 years. Overall, HIV prevalence is higher in women than men in most age groups [1]. More than 90% of the children who acquired HIV infection in 2011 live in sub-Saharan Africa. There, the number of children newly infected fall by 24% from 2009 to 2011 [2]. In 2011, the world made additional progress in advancing towards the 2015 goal, generating significant confidence in the feasibility of eliminating new infections among children by 2015 [2]. HIV infected women seen in the pre-treatment era had 27% transmission rates, falling to only 1.9% in infants of women on highly active anti-retroviral therapy (HAART). Mortality rate after HAART introduction is significantly lower than the period before the availability of such therapy [3]. Significant reduction in mother-to-child transmission (MTCT) of HIV has been achieved with the introduction of PMTCT in many countries of the world [4]. For example in South Africa [5] study revealed an infection rate of 2.7% was observed and in Nigeria [6, 7] it has fallen from 22.5% without PMTCT to 9.6% with mother having received PMTCT. HIV prevalence among HEI in Addis was 6.8% at age of six weeks [8]. Other studies found in the range of 5% [9] to 9.6% with PMTCT and 10.5% without PMTCT [6, 9]. Studies revealed that 64% took zidovudine (AZT) while 33% got ART and 73% of infants ingested medication at birth [10].

The key factors that impact HIV infection among HIV Exposed Infants (HEI) are maternal prophylaxis of either combination ART or single dose nevirapine (sdNVP); mode of delivery, maternal age, and by feeding method [5, 6, 9, 11]. In order to correctly inform parents/caregivers of infant infection status and link HIV-infected infants to care and treatment, retention of both mothers and their infants is both crucial and challenging to the health system. Substantial implementation barriers, as well as personnel and infrastructure requirements, exist at each step in the cascade. Even with the highest reported levels of uptake, nearly half of HIV-infected infants may not complete the cascade successfully [7]. In South African study mortality rate was 1.7% (95% CI: 0.6% to .3%) and there were 94 (36.2%) lost to follow ups (LTFUs) by six months [5]. Among 2477 HIV-exposed children registered for care by the United States Agency for International Development-Academic Model Providing Access To Healthcare partnership PMTCT program, 31 of 2477 infants (1.3%) were dead and 183 (7.4%) were lost to follow-up by 3 months [9]. Mother-infant pair enrolment in the same facility, early antenatal care (ANC) attendance and the infant’s father being tested and knew their HIV result were major predictors of infants adhering to treatment and follow up [5, 8, 10]. The study aimed to determine the proportion of HIV infection, identify associated factors with HIV infection and linkage of HEI to chronic care in Amhara Region.

## Methods

**Study design:** A cross-sectional study employing primary and secondary data was used among HEI in Amhara Region, Northern Ethiopia.

**Setting:** The study was conducted in selected health centers and hospitals in Amhara Regional State. The referral hospitals included Felegehiwot, Gondar, Debre Markos, and Dessie. The health centers are also situated in the same sites as the hospitals and are Gondar poly clinic, Debre Markos Health center, and Dessie health centers.

**Population:** All mother-infant pairs in Amhara region were the source population. Mother-infant pairs in Amhara region residing in catchment areas of the selected health center/hospitals were the study population. Infants and young children aged 2 years and below; mothers or care takers of infants and young children residing in the selected areas; mothers and HIV exposed infants who had been tested for HIV and knew their status; infant-mother/caretaker pairs who had at least two follow-up data were included. Children referred for chronic care and/or further therapy to other institution and second and third born infants from multiple births were excluded from the study.

The dependent variables of the study were HIV infection, linkage to chronic care while explanatory variables of the study included: socio-demographic variables of mother/ caretaker (age, sex, residence, educational status, relation with child, monthly income, and number of pregnancies), and infant/child: (age, sex, presence of parents, and length of follow up), clinical care characteristics(gestational age at PMTCT start, place of delivery, mode of delivery, vaccination status of child, prophylaxis for child, any illness during pregnancy, gestational age at first visit, maternal sepsis, preterm labor, maternal mortality). Other variables are stage of HIV disease in mother, duration of HIV infection, CD4+ count of mother, viral load of mother, parity, mode of delivery, infant birth weight, sex of infant and feeding modality.

**Operational definitions:** *HIV Exposed Infant* was defined as an infant born to a mother with confirmed HIV infection. *Linkage to chronic care* was regarded as an infant born to HIV infected mother or a mother who gave birth to HEI *that is* referred to chronic care clinic for further follow up.

### Sample size calculation and sampling techniques

Sample size was calculated using formula for single population proportions with the following assumptions: Z = 1.96, standard normal distribution at 95%confidence level, p = 6.8% (proportion of HEI who are HIV infected at six months using DNA/PCR) [8], d = 3% (margin of error), and Design effect = 2; and 10% non-response rate. The final sample size was 524.

A multistage random sampling was employed to select HEI - mother pairs. At the first stage of sampling, a simple random sampling technique was applied using lottery method to identify health centers and hospitals. The second stage of sampling was selection of infant-mother pairs using a systematic random sampling technique from selected health centers and hospitals among infants and mothers coming for chronic care follow up using the daily attendance list in the clinic. This was implemented guaranteeing the proportional infant mother pairs in each facility as per the client flow.

### Data collection procedures

A pre-tested, structured questionnaire developed in English and translated to Amharic that contained socio-demographic characteristics, family conditions, HIV status, and clinical care characteristics was used to collect quantitative data on infants and mothers. English version data extraction formats were used to retrieve some data from the charts of infants/children and their mothers. All the questionnaires and formats were pretested and corrected before the study resumed.

Data were collected by nurses and health professionals working outside of the facility under study who were given two days training. Daily supervision during the data collection period has been undertaken to maintain the quality of data and filled questionnaires were checked for completeness and accuracy.

### Data quality control

To maintain the quality of data, the questionnaire was pre-tested and structured. Data collectors were adequately trained on interview techniques and measurements. Standard measuring tools for weight and height measurements were used. Completed questionnaires were checked daily. Supervision by the investigators throughout the data collection was carried out. Data were entered in to Epi Info software version 7.0 to control for error during data entry.

### Statistical analysis

Data were entered, and cleaned using Epi Info version 7 software for windows and analyzed using SPSS 20 for windows. Descriptive statistics were used to present the socio-demographic characteristics, pregnancy related conditions, and magnitude of HIV infection. A chi-square test was used to identify determinants of HIV infection among HEIs. A p-value less than 0.05 was considered a statistically significant association.

### Ethical issues

Ethical approval was obtained from Institutional Review Board of the University of Gondar. Letter of permission was obtained from the School of Medicine, University of Gondar. Permission to pursue study was obtained from each facility. Informed consent was obtained from each mother/ guardian before the start of interview. The questionnaires were anonymous and no names of children or mothers were used. The data collected from each participant were kept confidential and locked. Any infant or mother had the right to withdraw at any point during data collection.

## Results

### Socio-demographic characteristics of women

A total of 484 mother-infant pairs were studied giving a response rate of (92.4%). Nearly 20 questionnaires were excluded due to significant missing information. Most (91.7%) women were from urban areas. More than half (56.2%) of them were in the age range of 20-29 years. Three-forth (75.2%) of mothers were married and two hundred eighty five (58.9%) were house wives while nearly half (46.9%) of women achieved educational level of secondary or above. One-third (33.7%) of women have delivered 5 or more times (Table 1).

**Table 1:** Demographic characteristics of HIV positive mothers in Amhara region, May-July 2013

Characteristic	Number	Percent
**Age of mother**		
< 20	3	.6
20-29	272	56.2
30-39	199	41.1
> 40	10	2.1
**Residence**		
Urban	444	91.7
Rural	40	8.3
**Marital Status of mother**		
Single	59	12.2
Married	364	75.2
Divorce	46	9.5
Separated	4	0.8
Widowed	11	2.3
**Occupation of mother**		
Gov't/self employed	110	22.7
Farmer	22	4.5
Housewife	285	58.9
Other	67	13.8
**Number of Births**		
Only One	163	33.7
2-4	158	32.6
5 or more	163	33.7
**Educational level of mother**		
Unable to read/write	140	28.9
Read and write only	39	8.1
Elementary	78	16.1
Secondary	183	37.8
College	44	9.1
**Religion of mother**		
Orthodox Christian	417	86.2
Muslim	61	12.6
Other	6	1.2

### Characteristics of infants

A total of 484 children (50.2% females and 49.8% males) were included in the study. Majority (80.8%) of children were 6-12months old with the mean (+s.d.) age of 10.9 (+ 9) months. The mean birth-weight of infants was 2.9 (+ 0.6) kilograms. More than two-thirds of infants lie in the range of 2.5-4.0 kilograms (Table 2).

**Table 2:** Basic characteristics of HIV exposed infants in Amhara region, Northwest Ethiopia, May-July 2013

Infant characteristics	Number of infants	Percent
**Sex of infant**		
Female	243	50.2
Male	241	49.8
**Age of infant/child**		
6-12 months	391	80.8
13-18 months	93	19.2
**Length/height of child**		
< = 50 cm	7	1.4
50-100cm	475	98.1
100-150cm	2	.4
**Birth-weight of infant [grams]**		
< 1500	19	3.9
1500-2499	30	6.2
2500-3999	353	72.9
> = 4000	20	4.1
**Prophylaxis to infant**		
sd NVP	274	56.6
sd NVP + AZT(1week)	23	4.8
sd NVP +single dose AZT	3	.6
AZT (1 week)	33	6.8
Other	106	21.9
Not given	45	9.3
**HIV status of infant**		
Infected	60	12.4
Negative	424	87.6
**WHO stage [n = 13]**		
1	4	30.8
2	5	38.4
3	4	30.8

### Pregnancy and delivery characteristics

Majority (94.4%) of women had antenatal care follow up for the current pregnancy and the same proportion (94.0%) of women delivered in health institutions. Most (94.4%) said that they had no problems during pregnancy while the rest reported to have some form of illness or pregnancy related problem. Spontaneous vertex delivery (SVD) constituted for most of the modes of deliveries where they had delivered without episiotomy (81.6%) or with episiotomy (11.6%). About 6.8% delivered through cesarean section (C/S). Among all, 8.5% had no any prophylaxis during pregnancy or delivery (Table 3).

**Table 3:** Pregnancy, delivery and maternal prophylaxis among HIV infected women in Northwest Ethiopia, May-July 2013

Pregnancy and delivery services	Number	Percent
**Antenatal care for this pregnancy**		
No ANC	27	5.6
Attended ANC	457	94.4
**Place of delivery**		
Health Institution	455	94.0
Home	29	6.0
**Health problems during delivery**		
No problems during delivery	457	94.4
Problem during pregnancy	27	5.6
**Mode of delivery**		
SVD	395	81.6
SVD with Episiotomy	56	11.6
Cesarean Section	33	6.8
**PMTCT**		
HAART	373	77.1
AZT +sdNVP	67	13.8
sdNVP	3	.6
No prophylaxis	41	8.5
**ART during pregnancy**		
Uninterrupted 1^st^ line	295	61.0
With interruptions 1^st^ line	14	2.6
d4T/3TC	23	4.8
LPV/r	24	5.0
3TC/ABC	16	3.3

### Linkage to chronic care of infants and children

About 94.2% of infants and women were linked to chronic care follow-up sometime after the diagnosis while 5.8% were not linked during the required time and the same 94.2% were given appointment after delivery for arrangement of further follow up. However, the appointment dates were not suitable for 16.9% of women who have been given appointments for follow-up. The reach of care for the mother was either very easy or easy for 55.4% and 41.7% of women respectively, while it was difficult or very difficult for 2.5% and 0.4% of women respectively. Fifty six (11.6%) of women declared that they were not sent to other clinics in the same facility when they were required to attend (Table 4).

**Table 4:** Chronic care linkage of infants and women, in Northwest Ethiopia, May-July 2013

Chronic care linkage characteristics	# of woman-child pairs	Percent
**Linkage to chronic care**		
Not linked	28	5.8
Linked	456	94.2
**Appointment date given after delivery**		
No	28	5.8
Yes	456	94.2
**Suitability of appointment dates ever given**		
Not suitable	82	16.9
Suitable	402	83.1
**Ease of reaching care for mother**		
Very easy	268	55.4
Easy	202	41.7
Difficult	12	2.5
Very difficult	2	0.4
**Ease of reaching child clinic**		
Very easy	267	55.2
Easy	206	42.6
Difficult	10	2.1
Very difficult	1	0.2
**Comfort of service during follow ups**		
Uncomfortable	26	5.4
Comfortable	458	94.6
**Timeliness of ART**		
Not timely	25	5.2
Timely	459	94.8
**OI prophylaxis**		
No	20	4.1
Yes	464	95.9
**Sent to other clinic in same facility when needed**		
No	56	11.6
Yes	428	88.4

### Infant HIV infection and correlates

Among 484 infants served in all the facilities studied, the level of HIV infection was 12.4%. There is no difference between males and females with regards to HIV infection (P = 0.81). At the same time, residence (rural or urban) has no association with HIV infection (p = 0.3). Antenatal care attendance has a significant impact on the occurrence of HIV infection among HEI (p < 0.0001). Delivering in health institution also resulted in lesser HIV infections (p < 0.005) as is having no problems during delivery (p < 0.028) and mode of delivery (p < 0.032). Provision of both infant (p < 0.0001) and maternal (p < 0.0001) prophylaxis have a highly significant association with prevention of HIV infection among HIV exposed infants (Table 5).

**Table 5:** Determinants of HIV infection among HEI in Amhara region, Northwest Ethiopia, May July 2013

Maternal/infant characters	Infant's HIV status		Chi-square (*p-value*)
	Infected	Negative	
**Sex of infant**			
Female	31	212	0.6 (*0.81*)
Male	29	212	
**Residence of Mother**			
Urban	53	391	1.05(*0.3*)
Rural	7	33	
**ANC for current pregnancy**			
No ANC	15	12	*<0.0001* (fisher's test)
Attended ANC	45	412	
**Place of delivery**			
Health Institution	51	404	0.005 (fisher's test)
Home	9	20	
**Health problems during delivery**			
No problems during delivery	53	404	4.8 (*0.028*)
Problem during pregnancy	7	20	
**Mode of delivery**			
SVD	43	352	6.9 (*0.032*)
SVD with Episiotomy	13	43	
C/S	4	29	
**PMTCT**			
HAART	37	336	41.4 *(<0.0001*)
sdNVP or AZT + sdNVP	5	65	
No prophylaxis	18	23	
**Infant prophylaxis**			
sd NVP	27	250	45.9 (*<0.0001*)
sd NVP + AZT 1 week	7	16	
AZT 1 week	2	31	
No prophylaxis	18	27	
Other prophylaxis methods^+^	6	100	

## Discussion

This research identified the level of HIV infection among HEI and the extent to which linkage to chronic care has been implemented in the region. A sample of health center and hospitals were included in the study. The level of HIV infection (12.4%) among HEI in Amhara region has been still high despite a major intervention by the Ministry of Health with a national logo of ?no child shall be born HIV infected?. A study in Addis Ababa reported a 6.8% infection rate; is lower than this study. One of the reasons is the study in Addis Ababa included the result from DNA/PCR at six months while this study included all until the age of 18 months that might have increased the rate as infections after six months of age are detected [8]. This is much higher than studies in other settings [9] where 5% were HIV infected. This might be partly due to the high burden of HIV in this country. The other study had reported 7.4% losses-to-follow up and 1.3% deaths which were not included in the analysis as HIV status were not known. Those LTFUP might have been infants with HIV infection thereby underestimating the HIV prevalence.

However, this was lower than findings from Nigeria [6] where the overall average infection rate was 22.5% which might be due to inclusion of many mothers with no PMTCT as compared to our study where the PMTCT coverage was high. Similar higher rates were also reported from other studies [4].

In this study antenatal care follow-up has a positive impact in preventing transmission of HIV among HEI (P < 0.001). This is because antenatal care is an opportunity to get maternal prophylaxis or ARVs that significantly reduces vertical transmission. Other studies in Zambia showed a significant reduction of vertical transmission of HIV [11]. The place of delivery is also found to be significantly associated with HIV infection in HEI again due to an increased opportunity for babies delivered in health facilities to get infant prophylaxis thereby protection from infection. The mode of delivery has also impact on infection as some operative deliveries like episiotomy increase the risk of infection and the C/S reduces duration of labor and hence lesser risk of infection.

The decreased risk of HIV infection among HEI in those who were on either infant or maternal prophylaxis shows the health system need to reach all women for PMTCT and encourage institutional delivery accompanied by proper provision of follow up and treatment.

## Conclusion

The level of HIV infection among HEI in Amhara region remains high despite all interventions. Maternal antenatal care, institutional delivery, cesarean delivery, and provision of infant and maternal prophylaxis were positive predictors of preventing HIV infection among HEI. A significant portion of infants and mothers identified as HIV exposed or infected respectively were left unlinked to the chronic HIV care. This leads to missed opportunity to prevent HIV infection in exposed infants and to provide treatment in HIV infected women.

The health facilities shall encourage antenatal care that entails increased institutional delivery, high uptake of PMTCT and infant prophylaxis to reduce the vertical transmission of HIV infection and meet national targets. Health facilities shall reorganize the care system from labor ward to the chronic care unit to effectively reach each woman to continue HIV care and provide testing and subsequent therapy of exposed children.

### What is known about this topic

- Prevalence of HIV infection in exposed infants before the scale up of PMTCT;
- The key factors that impact HIV infection among HIV Exposed Infants (HEI); maternal prophylaxis; mode of delivery, maternal age, and feeding method.

### What this study adds

- Prevalence of HIV infection among exposed infants after PMTVT scale up;
- Linkage to chronic care of HIV infected infants;
- Place of delivery, antenatal care and complication during delivery as correlates of HIV infection in exposed infants.

## References

[1] Central Statistical Agency [Ethiopia] and ICF International (2012). Ethiopia Demographic and Health Survey 201.

[2] Joint United Nations Programme on HIV/AIDS (UNAIDS) (2012). UNAIDS Report on the global AIDS epidemic.

[3] Noel F, Mehta S, Zhu Y (2008). Improving outcomes in infants of HIV-infected women in a developing country setting. PLoS one..

[4] Harjot K, Nikhil G, Aarti K (2011). High rates of all-cause and gastroenteritis related hospitalization morbidity and mortality among HIV-exposed Indian infants. BMC Infectious Diseases.

[5] Terusha C, Stephen K, Janet G, Tamaryn L, Lisa M, Marie-Louise N (2012). A retrospective study of Human Immunodeficiency Virus transmission, mortality and loss to follow-up among infants in the first 18 months of life in a prevention of mother-to-child transmission programin an urban hospital in KwaZulu-Natal, South Africa. BMC Pediatrics.

[6] Abayomi J, Niyi A (2011). Outcome of PMTCT services and factors affecting vertical transmission of HIV infection in Lagos, Nigeria. HIV and AIDS Review..

[7] Andrea L, Ji-Eun P, Lynn R, Kenneth A, Rochelle P, Valeriane L (2011). Early infant HIV-1 diagnosis programs in resource limited settings: opportunities for improved outcomes and more cost-effective interventions. BMC Medicine.

[8] Mulatu B, Frida E, Addisalem A (2011). Prophylactic treatment uptake and compliance with recommended follow up among HIV exposed infants: a retrospective study in Addis Ababa, Ethiopia. BMC Research Notes.

[9] Nyandiko W, Otieno-Nyunya B, Musick B (2010). Outcomes of HIV-exposed children in western Kenya: efficacy of prevention of mother to child transmission in a resource-constrained setting. J Acquir Immune Defic Syndr..

[10] Alemnesh H, Sven G, Mitike M, Karen M, Odd M (2011). Current status of medication adherence and infant follow up in the prevention of mother to child HIV transmission programme in Addis Ababa: a cohort study. Journal of the International AIDS Society..

[11] Torpey K, Mandala J, Kasonde P (2012). Analysis of HIV early infant diagnosis data to estimate rates of perinatal HIV transmission in Zambia. PLoS One.

